# Frailty measures in the critically ill: are we approaching a critical age? A systematic review

**DOI:** 10.1186/cc13240

**Published:** 2014-03-17

**Authors:** R Pugh, DL John, C Thorpe, C Subbe

**Affiliations:** 1 Glan Clwyd Hospital, Bodelwyddan, UK; 2 Ysbyty Gwynedd, Bangor, UK

## Introduction

As the general population ages, the proportion of critically ill patients in whom frailty may adversely affect outcome is likely to rise. The aim of this systematic review was to evaluate performance of frailty measures in predicting ICU, hospital and long-term outcomes following intensive care admission.

## Methods

We performed a literature search for original studies in: EMBASE, MEDLINE, Web of Knowledge, Cochrane database of systematic reviews, and Database of Abstracts of Reviews of Effects, using the terms ‘frailty', ‘frail elderly', ‘critical care', ‘critically ill', ‘critical illness' and ‘intensive care'. Our study inclusion criteria were that the study: included patients cared for in intensive care, captured data relating to premorbid frailty, and reported ICU, hospital and/or longterm outcome data.

## Results

Initial searches identified 606 reports, of which, following review of abstracts and removal of duplicates, 66 full-text papers were evaluated. Of these, 11 met inclusion criteria. A further 19 papers that met inclusion criteria were identified from relevant review articles and reference lists. There was huge variation in populations studied, methodology, frailty measures utilised and reported outcome measures. Of the 30 included studies, 11 studies evaluated patients undergoing major (including cardiothoracic) surgery, two studies specifically assessed patients with pneumonia, one study investigated patients in a burns ICU and one study restricted its investigation to former nursing home residents. The most commonly used measures of frailty were: measures of Activities of Daily Living (*n *= 9), which predicted need for long-term institutional care, 30-day, 90-day, 6-month and 12-month mortality; Clinical Frailty Score [1] (*n *= 5), which predicted ICU mortality, hospital mortality, 12-month mortality, quality of life and functional dependence; and Knaus score [2] (*n *= 4), which predicted ICU mortality, hospital and 12-month mortality.

## Conclusion

Measures of frailty appear to predict mortality and functional dependence following critical admission across a wide range of clinical conditions. However, comparative data regarding the relative accuracy and reliability of frailty measures in the intensive care population are currently deficient.
